# *Cryptosporidium* spp. Are Associated with *Giardia duodenalis* Co-Infection in Wild and Domestic Canids

**DOI:** 10.3390/ani14233484

**Published:** 2024-12-02

**Authors:** Maira Mateusa, Aivars Cīrulis, Maija Selezņova, Dārta Paula Šveisberga, Margarita Terentjeva, Gunita Deksne

**Affiliations:** 1 Institute of Food Safety, Animal Health and Environment “BIOR”, LV-1076 Riga, Latvia; aivars.cirulis@bior.lv (A.C.); maija.seleznova@bior.lv (M.S.); darta.sveisberga@bior.lv (D.P.Š.); margarita.terentjeva@lbtu.lv (M.T.); gunita.deksne@bior.lv (G.D.); 2 Faculty of Veterinary Medicine, Latvia University of Life Sciences and Technologies, LV-3004 Jelgava, Latvia; 3 Faculty of Medicine and Life Sciences, University of Latvia, LV-1004 Riga, Latvia; 4 Department of Residency, Riga Stradiņš University, LV-1007 Riga, Latvia

**Keywords:** domestic dog, red fox, raccoon dog, protozoans, Latvia, zoonosis, prevalence

## Abstract

*Cryptosporidium* spp. and *Giardia duodenalis* are zoonotic food- and water-borne protozoa of veterinary importance. They are one of the main causes of diarrhea in domestic dogs (*Canis familiaris*). Wild canids, such as red foxes (*Vulpes vulpes*) and raccoon dogs (*Nyctereutes procyonoides*), can act as natural reservoirs for these parasites. The aim of this study was to estimate the prevalence of these pathogens and analyze possible factors that may affect the infection in wild and domestic canids. A total of 373 domestic dog, 219 red fox, and 78 raccoon dog fecal samples were collected and tested for *Cryptosporidium* spp. and *G. duodenalis* presence with direct immunofluorescent staining. The dog owners filled a questionnaire on the dog’s habits and lifestyle to identify and analyze potential factors that may affect the occurrence of these parasites in dogs. *Cryptosporidium* spp. were found in 16.3%, 28.3%, and 28.2% and *G. duodenalis* in 0.7%, 27.4%, and 30.8% of the examined dogs, red foxes, and raccoon dogs, respectively. The highest prevalence was observed in puppies—15.4% and 18.5% for *Cryptosporidium* spp. and *G. duodenalis*, respectively. In all three canid species, infection with *Cryptosporidium* increases the risk of co-infection with *Giardia* and vice versa.

## Simple Summary

*Cryptosporidium* and *Giardia* are single-cell parasites that can infect both animals and humans, causing diarrhea and risks to public health. Both parasites can be found in nearly all mammals, including domestic dogs, red foxes, and raccoon dogs, which can act as natural carriers and spread them through direct contact, food, and the environment. This study aimed to understand how common these protozoans are in Latvia and what factors influence their occurrence in domestic and wild canids. We collected fecal samples from 373 domestic dogs, 219 red foxes, and 78 raccoon dogs and tested for the presence of *Cryptosporidium* and *Giardia duodenalis*. Red foxes and raccoon dogs had higher prevalence of both parasites than domestic dogs. Puppies showed higher infection rates with both parasites. All infected canids were more likely to have both parasites simultaneously. This research highlights the need for responsible pet care and a potential zoonotic risk for humans.

## 1. Introduction

*Cryptosporidium* spp. (Tyzzer, 1907) and *Giardia duodenalis* (Stiles, 1902) are important zoonotic protozoans that can cause diarrhea of varying severity in the vast majority of vertebrates, including canids—domestic dogs (*Canis familiaris*), red foxes (*Vulpes vulpes*), and raccoon dogs (*Nyctereutes procyonoides*)—as well as humans [1]. Currently, at least 44 *Cryptosporidium* species with 120 genotypes have been identified, with *C. canis* often reported in dogs and other canids [1,2]. However, numerous reports of the zoonotic *C. parvum* have also been recorded in dogs [3,4,5]. Of the eight *Giardia* species currently recognized, *G. duodenalis* is of particular interest in public health. There are eight assemblages (A–H), out of which assemblages A and B are zoonotic and can infect a vast range of mammals [2,6]. Canids are mainly affected by assemblages C and D, but there have also been sporadic reports of infections with assemblages A and B [6].

Cryptosporidiosis in domestic dogs is usually asymptomatic, but clinical signs, such as diarrhea, can be observed in younger dogs, especially if the animal is under chronic stress [7]. High infection rates are observed in three-, four-, and six-month-old dogs, with the infection declining in dogs above one year old [8,9]. The global estimated prevalence of *Cryptosporidium* in dogs reaches 8.0%, and in Europe, it can vary between 1.1% and 20.5% [10,11,12].

*Giardia* is one of the most commonly detected parasites in dogs; its worldwide prevalence reaches 15.2%, with a higher prevalence observed in shelter dogs and dogs from kennels [13,14,15]. In Europe, however, *Giardia* prevalence can vary between 15% and 30.5% [12,16,17]. Likewise, the risk of giardiasis increases if dogs are placed in a shelter for a prolonged period of time [18]. In contrast to cryptosporidiosis, the clinical manifestation of giardiasis in dogs is more severe: abdominal pain, cramping, watery diarrhea, steatorrhea, and malabsorption [19,20]. Often, the course of the disease can be asymptomatic, chronic, or intermittent, complicating the diagnosis [19,20].

Scarce information is available on the clinical signs of both parasite infections in red foxes and raccoon dogs. In red foxes, *Cryptosporidium* prevalence varies from 2.2% to 3.2%, but *Giardia* prevalence ranges from 2.8% to 44.0% in various European countries, such as Norway, Bosnia and Herzegovina, and Sweden [21,22,23]. In raccoon dogs, the prevalence of *Cryptosporidium* is estimated to be 17.6% in Poland [24]. Additionally, wild canids can act as possible reservoirs for both pathogens for domestic dogs and humans due to the zoonotic *C. parvum* and *G. duodenalis* assemblages A and B [21,25,26].

Identifying the prevalence of these pathogens in domestic dog, red fox, and raccoon dog populations is important to establish whether wild and domestic canids also pose such infection risks to humans. The aim of this study is to estimate the prevalence of *Cryptosporidium* and *G. duodenalis* in canids of Latvia—domestic dogs, red foxes, and raccoon dogs—and analyze factors affecting both infections in canids.

## 2. Materials and Methods

### 2.1. Study Area of Domestic Dogs

The necessary sample size was calculated using the OpenEpi v.2.3.1. open access program, assuming a 95% confidence interval (95%CI) and a 40% infection frequency in the domestic dog population [27]. The officially registered Latvian domestic dog population of 132,750 was retrieved from the Agricultural Data Centre Republic of Latvia (accessed on 17 January 2020) [28].

To reach a larger target audience, owners, veterinarians, and shelter owners were invited to participate in the study either through private contact or by responding to social media advertisements. Questionnaires, together with sterile collection containers, were provided to the participating veterinary clinics, shelters, and client centers of the Institute of Food Safety, Animal Health and Environment “BIOR” (Institute “BIOR”). It was recommended that fecal samples be collected for three days in a row. After the collected samples and the filled-out questionnaires arrived at the laboratory, they were labeled, fecal consistency was noted (liquid, soft, or formed), and the samples were stored at 4 °C until further testing.

### 2.2. Questionnaire for Domestic Dogs

A questionnaire was designed to gather general information about the dogs (breed, sex, age, living conditions), their daily activities (walks in the city, parks, meadow, and forest), health (general information about diarrhea), deworming (frequency of medication and deworming medicine used), feeding (raw, commercial, home-cooked, game meat), contact with other animals (including through home slaughter of livestock) (Supplementary File S1). The dog population was stratified by age: puppies (up to 2 years), adults (2 to 7 years old), seniors (8 to 11 years old), and geriatric (above 12 years old) [29]. The owner filled a questionnaire in Latvian.

### 2.3. Study Area of Wild Canids

Fecal samples from red foxes and raccoon dogs were collected during the Latvian State program for the Control and Eradication of Rabies organized by the Food and Veterinary Service [30]. Carcasses of hunted foxes and raccoon dogs were delivered to the scientific Institute “BIOR” by hunters, mainly from the eastern part of Latvia, together with additional information about the age, based on dental wear, of the animal (if recorded) and the location (forestry) of where the animal was obtained. Feces from the gastrointestinal tract were collected and stored in a clean container, labeled, and stored at 4 °C until further testing.

### 2.4. Sample Preparation

One gram of fecal sample was prepared in a clean 15 mL centrifuge tube (SARSTEDT, Nümbrecht, Germany), using equal parts of a saturated sodium chloride (NaCl; density 1.18) solution and distilled water and submitted to a one-minute centrifugation step at 1560× *g*. In the next step, the supernatant was poured into a clean 50 mL centrifuge tube (SARSTEDT, Nümbrecht, Germany), and distilled water was added to the 45 mL mark. Afterward, the 50 mL tube was centrifuged thrice for 10 min at 1560× *g*. Between these steps, the top layer was poured out till the 5 mL mark; the sediment was vortexed, and distilled water was added again, resulting in 2 mL of purified and concentrated sediment [31,32].

For immunofluorescence staining, according to the manufacturer’s instructions, 10 μL of thoroughly mixed sample was stained with fluorescein isothiocyanate (FITC)-labelled anti-*Cryptosporidium*/*Giardia* monoclonal antibodies (AquaGlo^TM^, Waterborne Inc., New Orleans, LA, USA). For each batch, the positive control provided in the AquaGlo^TM^ stain kit was added once to assess the quality of the stain. All bright green (oo)cysts with typical morphology and size were counted, and the total count was multiplied by 200 to calculate the total (oo)cysts per gram of feces [32].

### 2.5. Data Analysis

Descriptive statistics (medians and means) for oocysts (OPG) and cysts per gram (CPG) were calculated for age groups, breed, and sex. The 95% CI was calculated using the Mid-p exact test using the OpenEpi v.2.3.1. open access program [27].

Standard deviation (SD) was calculated for the animal ages. A two-tailed Mid-p exact test was used to calculate the *p*-value for differences between purebred and mixed dog species, as well as sexes; *p* < 0.05 was considered statistically significant. The chi-square test was used to calculate the *p*-value between dog age groups, and *p* < 0.05 was considered statistically significant. An animal was considered positive if its fecal sample had at least one *Cryptosporidium* or/and *Giardia* (oo)cyst present.

Using QGIS (v.3.36.0-Maidenhead), a map displaying the canid sampling locations and the infection status of *Cryptosporidium* spp. and *G. duodenalis* was created [33].

Forward and backward logistic regression model selection was performed using R (v.4.3.2), based on the lowest Akaike information criterion (AIC and AICc) values (from performance package, function performance) for generalized linear models (GLMs) with the family set to binomial [34,35]. The final models had to align with the assumptions (multicollinearity was checked for with function vif from the car package) and included factors that appeared to be significant in single-factor logistic regression analyses to assess if they remained significant [36]. The response (dependent) variable was testing either positive or negative for *Cryptosporidium* spp. or *G. duodenalis*. The significance of each independent variable was assessed with the function summary or Anova function with type III sum of squares from the car package [36]. A result was considered statistically significant at *p* < 0.05, while *p* < 0.1 was considered to indicate a trend and was further investigated. Tjur’s coefficient of discrimination (Tjur’s R^2^) was calculated from the performance package function performance to assess how much a particular model explained the probability of having or not a particular infection.

## 3. Results

### 3.1. Overall Prevalence of Cryptosporidium spp. and G. duodenalis in Dogs

**Figure 1:**
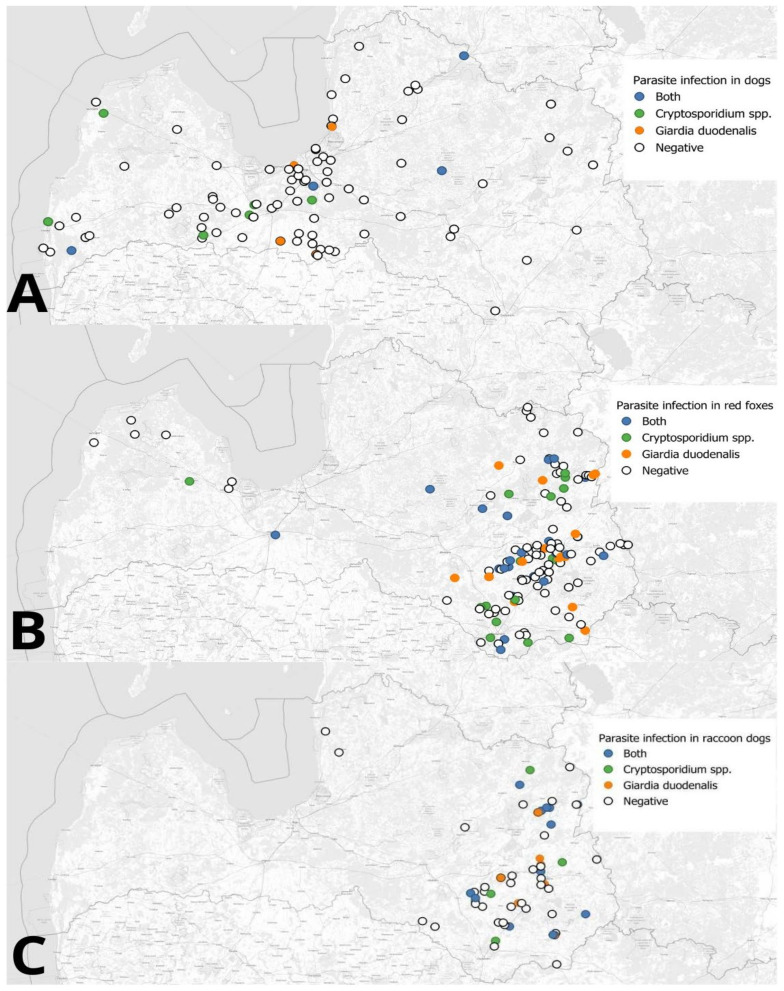
Fecal sample collection sites in Latvia for canids and positive results of *Cryptosporidium* spp. and *G. duodenalis*. (**A**) Domestic dogs; (**B**) red foxes; (**C**) raccoon dogs.

From February 2020 to June 2023, a total of 373 fecal samples from domestic dogs with a mean age of 5.4 years (SD ± 4.1; min 0.2; max 17; median 4) were collected (Figure 1).

In total, 61 dogs (16.3%; 95%CI: 12.9–20.5) were positive for one or both parasites. *Cryptosporidium* spp. were detected in 9.9% (n = 37; 95%CI: 7.2–13.4) of the animals, with the highest prevalence observed in puppies (Table 1). The mean OPG was 5400 (min 200; max 30′800; median 800), with the highest OPG observed in geriatric dogs (Table 1). No significant statistical significance was observed for *Cryptosporidium* spp. between age groups, purebred and mixed breeds, and sexes (*p* > 0.05) (Table 1).

*G. duodenalis* was found in 10.7% (n = 40; 95%CI: 8.0–14.3) of the examined dogs, with a mean CPG of 35,368 (min 200; max 393,600; median 4200). Similarly to *Cryptosporidium*, puppies had the highest prevalence of giardiasis (Table 1). Male dogs had a significantly higher prevalence of *Giardia* than female dogs (Table 1 and Table 2), with 14.7% and 6.6% prevalence, respectively.

Co-infections were identified in 4.3% (n = 16; 95%CI: 2.6–6.9) of all the examined dogs. On average, the dogs that were positive with both parasites excreted 3263 (median 800) OPG and 25,012 (median 4800) CPG. The mean age of the dogs infected with both parasites was 4.4 years (SD ± 3.9, median 3.5).

### 3.2. Overall Prevalence of Cryptosporidium spp. and G. duodenalis in Wild Canids

A total of 219 red fox fecal samples were collected from 2020 to 2023 (Figure 1). Age in years was recorded for 169 foxes, with an average age of 2.1 years (SD ± 0.81, min 1; max 5).

In total, *Cryptosporidium* spp. prevalence was 28.3% (n = 62; 95%CI: 22.7–34.6), and *G. duodenalis* prevalence was 27.4% (n = 60; 95%CI: 21.9–33.7). Both parasites were detected in 16.4% of all examined red foxes (n = 36; 95%CI: 13.8–24.8). The average OPG was 5364 (min 200; max 62,800; median 1200), and CPG was 3133 (min 200; max 47,600; median 700).

A total of 78 fecal samples from raccoon dogs were collected during 2020–2023 (Figure 1). Age in years was recorded for 55 raccoon dogs, with an average age of 2 years (SD ± 0.64, min 1; max 4.5).

In total, *Cryptosporidium* spp. were detected in 28.2% (n = 22; 95%CI: 19.4–39.1), and *G. duodenalis* in 30.8% (n = 24; 95%CI: 21.6–41.7) of the raccoon dogs. Both parasites were detected in 20.5% (n = 16; 95%CI: 12.9–30.9) of the raccoon dogs. The average OPG was 6072 (min 200; max 51,400; median 1400), and CPG was 15,082 (min 200; max 224,000; median 1200).

### 3.3. Models

#### 3.3.1. *Cryptosporidium* spp. and *G. duodenalis* in Domestic Dogs

The final model for *Cryptosporidium* spp. in domestic dogs with the lowest AIC values (AIC: 129.804, AICc: 130.094) was established by forward selection based on a single dependent variable model with *p* < 0.1. The risk factors included in the model were co-infections with *G. duodenalis*, home-based slaughter of livestock (chickens, ruminants, pigs), fecal consistency, and activity on a leash outside the city. Dogs with cryptosporidiosis were more likely to have *G. duodenalis* co-infection (Table 2), while the final model explained 19% (Tjur’s R^2^) of the probability of having *Cryptosporidium* spp. infection. Dogs with giardiasis were also more likely to have *Cryptosporidium* spp. co-infection (Table 2). Sex was a risk factor for domestic dogs with *G. duodenalis* infection (Table 2). The dog age did not affect either cryptosporidiosis or giardiasis (*p* = 0.15; *p* = 0.6, respectively).

#### 3.3.2. *Cryptosporidium* spp. and *G. duodenalis* in Red Foxes

For *Cryptosporidium* spp., the final model with the lowest AIC values (AIC: 162.443, AICc: 162.961) was retrieved by backward selection with the following factors affecting *Cryptosporidium* spp. in red foxes: co-infection with *G. duodenalis*, forestry, and old age (age in years; z = 0.7, Table 2). However, only co-infection with *G. duodenalis* and forestry were statistically significant (*p* < 0.05, Table 2). The final model explained 26% (Tjur’s R2) of the probability of having *Cryptosporidium* spp. infection.

For *G. duodenalis*, the final model with the lowest AIC values (AIC: 165.681, AICc: 165.925) was retrieved by forward selection based on single dependent variable models with *p* < 0.05, which included co-infection with *Cryptosporidium* spp., old age (age in years; z = 2.7), and their interaction, with all of them being statistically significant (*p* < 0.05, Table 2). In red foxes, the probability of *G. duodenalis* infection increased if the animal was already infected with *Cryptosporidium* spp. and was older (Table 2). The final model explained 25% (Tjur’s R^2^) of the probability of having *G. duodenalis* infection.

#### 3.3.3. *Cryptosporidium* spp. and *G. duodenalis* in Raccoon Dogs

In raccoon dogs, both models showed that if an animal was already infected with either *Cryptosporidium* or *Giardia*, then there was a higher probability of also being infected with the other pathogen (*p* < 0.01, Table 2), which was also observed in red foxes.

For *Cryptosporidium* spp., the final model with the lowest AIC values (AIC: 62.490, AICc: 64.240) was retrieved by backward selection, which included co-infection with *G. duodenalis*, younger age (age in years; z = −1.5), and forestry; only *G. duodenalis* co-infection appeared to be statistically significant (*p* < 0.001, Table 2). It was concluded that in raccoon dogs, *Cryptosporidium* spp. infection was positively affected only by *G. duodenalis* co-infection (Table 2), while the final model explained 27% (Tjur’s R^2^) of the probability of having *Cryptosporidium* spp. infection.

For *G. duodenalis*, the final model with the lowest AIC values (AIC: 53.576, AICc: 54.047) was retrieved by forward selection based on single dependent variable models with *p* < 0.08, which included *Cryptosporidium* spp. and younger age (age in years; z = −0.2), with only *Cryptosporidium* spp. being statistically significant (*p* < 0.01, Table 2). It was concluded that in raccoon dogs, *G. duodenalis* infection was positively affected by *Cryptosporidium* spp. co-infection (Table 2). The final model explained 27% (Tjur’s R^2^) of the probability of having *G. duodenalis* infection.

## 4. Discussion

Both parasites were prevalent in the domestic dog population, with prevalence reaching 9.9% for *Cryptosporidium* spp. and 10.7% for *G. duodenalis*.

A high overall *Cryptosporidium* spp. prevalence was observed in dogs under two years old (puppies), which is consistent with other studies [11,37,38]. Even though there was no statistical significance for the difference between age groups, *Cryptosporidium* showed a tendency to affect younger animals due to their immature immune system—puppies acquire passive immunity via the colostrum that is provided by the bitch [39]. Geriatric dogs excreted high levels of oocysts in their feces, possibly due to advanced age-related health decline, decreased number of T-cells, and changes in the intestinal microbiota [40,41]. These changes reduce the body’s ability to fight pathogens, including parasites, increasing the probability of shedding oocysts for a prolonged period [40]. It is also worth mentioning that the small sample size of the geriatric dog group positive for *Cryptosporidium* should be considered, and the results should be interpreted cautiously.

Similarly to *Cryptosporidium*, the highest prevalence of *Giardia* and the highest cyst output were observed in puppies (Table 1). *Giardia* is one of the most commonly detected parasites in dogs [17,42,43,44]. Studies show that dogs under one year old are more likely to shed *Giardia* cysts, but no association between the presence of pathogens and changes in fecal consistency was observed [9,45,46,47]. Several studies have been focusing on *Giardia*-induced clinical signs in dogs; however, the results are inconclusive: in some, a correlation between *Giardia* and diarrhea was observed, whereas, in others, no such association between infection and diarrhea was found [44,48]. These differences could be explained by the diverse clinical manifestations of giardiasis—ranging from asymptomatic to severe diarrhea—and sampling strategies (sampling from seemingly healthy dogs versus that from dogs with gastrointestinal signs). Nevertheless, *Giardia* should be considered a possible cause of gastrointestinal problems, especially if intermittent diarrhea is present [17,44]. Before the new 2024 World Small Animal Veterinary Association (WSAVA) vaccination guidelines, it was recommended to socialize puppies only after the final core vaccines were administered, which was at around 16 weeks of age; hence, puppies may get infected with *Cryptosporidium* and *Giardia* right after they start to go outside and socialize with other dogs [49,50].

Co-infections with both parasites were mostly observed in adult dogs, who shed high amounts of (oo)cysts in the environment. Additionally, in the final models (Table 2), a significant association was observed between *Cryptosporidium* spp. and *G. duodenalis* co-infections, indicating that the presence of one infection increased the likelihood of the other. Even though there have been observations of *Giardia* co-infections with other parasites, such as *Toxocara canis* and *Isospora* spp., especially in young dogs, no direct statistical correlation has been observed [17,51,52].

Even though there are still uncertainties about whether dogs pose a risk for human infections, several studies have shown that dogs are positive for the zoonotic *C. parvum* or *G. duodenalis* assemblages A and B [3,4,5,6,47]. Additionally, there have been several reports of humans being infected with *C. canis* [37,53,54]. Therefore, personal hygiene measures should be upheld when working or living with dogs, even if the animal does not show clinical signs. Regular routine parasitological examinations to detect and, if needed, treat these animals to minimize environmental contamination and risk for humans should also be considered. It is not clear whether cryptosporidiosis in dogs increases the probability of giardiasis and vice versa; however, prioritizing the diagnostics of both parasites should be strongly considered. Additionally, field experts should educate dog and dog shelter owners and handlers about the importance of feces collection from the environment after defecation to minimize the parasite load in the environment [55,56].

For both parasites, higher prevalence was observed in male dogs; however, in the final model, sex appeared to be a significant factor only for *Giardia*. Other studies show that either female dogs have higher *Cryptosporidium* and *Giardia* prevalence, or sex has no correlation at all with these infections [9,57,58]. Some studies show that male dogs are at a higher risk of developing diarrhea, which could be explained by their increased roaming and sniffing behavior, compared to female dogs, which increases the risk of contact with pathogens, including parasites [59,60].

In the initial model, home slaughter of livestock and poultry appeared to be a factor that increased the likelihood of cryptosporidiosis in dogs, but the final model showed only a trend toward significance (Table 2). Ruminants, especially cattle, shed high amounts of *Cryptosporidium* spp. oocysts in the environment [61]. Therefore, it is possible that these dogs were coincidentally infected through contact with ruminants, and this is not a direct representation of the home slaughter of livestock and poultry. However, feeding by-products of animal origin to dogs can be a route of infection for various other pathogens [62]. Even though the questionnaire used to gather information from the dog owners was comprehensive, it was not validated, which could have introduced some variability in the responses.

A higher prevalence of both parasites was observed in red foxes and raccoon dogs compared to domestic dogs. A high prevalence of *G. duodenalis* has also been observed in red foxes from Sweden; however, prevalences as low as 4.5% and 2.2% have been observed in Romania and Croatia [21,26,63]. Red foxes are widespread in Latvia, and because they tend to roam in urban areas, they can also pose a risk for humans due to their potential carrying of the zoonotic *G. duodenalis* assemblages A and B [21,26,63]. Red foxes also carry several *Cryptosporidium* species that pose a zoonotic risk—*C. parvum*, *C. hominis,* and *C. canis* [25,64]. Limited information is available about both parasites in raccoon dogs in Europe; however, *G. duodenalis* assemblage D has been reported in Poland and Romania, and *C. canis* in Poland [65,66,67]. Similar to dogs, also in wild canids, both parasites seem to impact each other (Table 2).

An interesting pattern was observed regarding *G. duodenalis* infection, which appeared to significantly increase with the age of red foxes (Table 2). This was an unexpected finding due to the fact that *G. duodenalis* infection was previously reported from juvenile Swedish foxes [22]. The same pattern was observed for *Cryptosporidium* spp. infection; however, all the results were insignificant. This is an unusual finding, especially since previous studies have shown that both parasites are more commonly associated with higher infection rates in dogs under one year of age [11,12]. This pattern was also observed in our study for both domestic dogs and raccoon dogs (Table 1 and Table 2). In this study, all wild canids were above one year old, and although both parasites tend to affect younger animals, if the animal is subjected to chronic stress or has an accompanying disease, such as sarcoptic mange infection, both parasites can develop chronic manifestations that can last for several months, or the animals can acquire the infection later in life due to a compromised immune system [8,68]. However, it is worth noting that age determination was performed by the hunters who sent in the samples; therefore, the exact age of these animals may be different.

This study did not use molecular methods to confirm the zoonotic *Cryptosporidium* species and *G. duodenalis* assemblages; hence, the true zoonotic risk from domestic dogs, red foxes, and raccoon dogs in Latvia still needs to be determined.

## 5. Conclusions

There was a highly significant association between *Cryptosporidium* spp. and *G. duodenalis* co-infections in all the canid species studied, suggesting a possible interaction or shared risk factors between the two pathogens. Both infections are prevalent in Latvian domestic dogs, with the highest prevalence observed in puppies. Because *Cryptosporidium* and *Giardia* are likely to cause co-infection, routine testing for both parasites should be prioritized if diarrhea is present, especially if it is intermittent. The prevalence of both parasites in red foxes and raccoon dogs was higher than in domestic dogs. Therefore, they could be a potential infectious source for humans and dogs.

## Figures and Tables

**Table 1:** The overall prevalence of *Cryptosporidium* spp. and *G. duodenalis* in different domestic dogs by age, breed (purebred and mixed breed), and sex.

Parasite Species/ Factor	Sub-Factor	Total No. of Analyzed/ Infected Animals	Prevalence (95% CI)	Mean OPG/CPG	Median OPG/CPG	Min–Max OPG/CPG	*p*-Value
*Cryptosporidium* spp.							
Age group	Puppy	65/10	15.4 (8.8–25.2)	3680	2000	200–25,200	0.426
	Adults	193/16	8.3 (5.1–26.2)	5688	1000	200–25,000	
	Seniors	96/9	9.4 (4.4–15.7)	7533	2200	200–30,600	
	Geriatric	19/2	10.5 (1.7–32.6)	21,100	21,100	11,400–30,800	
Breed	Purebred	218/23	10.5 (7.1–15.4)	8100	2400	200–30,800	0.792
	Mixed	155/14	9.0 (5.3–14.7)	8160	3200	200–30,600	
Sex	Female	183/16	8.4 (5.4–13.8)	6109	2000	200–25,000	0.463
	Male	190/21	11.0 (7.2–16.4)	9679	2800	200–30,800	
*G. duodenalis*							
Age group	Puppy	65/12	18.5 (10.7–29.7)	39,650	24,800	400–157,800	0.162
	Adults	193/18	9.3 (5.9–14.3)	35,225	3900	200–33,000	
	Seniors	96/9	9.4 (4.8–17.1)	46,488	1600	200–393,600	
	Geriatric	19/1	5.3 (0.1–26.5)	NA	NA	NA	
Breed	Purebred	218/22	10.1 (6.7–14.9)	30,522	22,700	200–157,800	0.641
	Mixed	155/18	11.6 (7.4–17.7)	30,600	3200	200–36,600	
Sex	Female	183/12	6.6 (3.7–11.2)	27,250	8800	200–89,600	0.011 *
	Male	190/28	14.7 (10.3–20.5)	56,440	5300	200–393,600	

Abbreviations: CI—confidence interval; NA—not applicable; * *p* < 0.05.

**Table 2:** Analysis of deviance (type III tests) for binomial generalized linear models to test if the respective fixed effects (factors) affected the *Cryptosporidium* spp. or *G. duodenalis* infection status in domestic dogs, red foxes, and raccoon dogs.

Canid Species	Parasite	Factor	LR Chisq	Df	*p*-Value
Domestic dog	*Cryptosporidium* spp.	*G. duodenalis*	12.2	1	<0.001 ***
		Home slaughter of livestock	3.3	1	0.07
		Fecal consistency	1.6	1	0.2
		Activity outside the city—on leash	2.7	1	0.1
	*G. duodenalis*	*Cryptosporidium* spp.	28.3	1	<0.001 ***
		Sex	5.4	1	0.02 *
		Activity outside the city—on leash	3.5	1	0.06
Red fox	*Cryptosporidium* spp.	*G. duodenalis*	32.1	1	<0.001 ***
		Forestry	11.2	3	0.01 *
		Age (years)	0.5	1	0.5
	*G. duodenalis*	*Cryptosporidium* spp.	17.2	1	<0.001 ***
		Age (years)	7.6	1	0.006 **
		*Cryptosporidium* spp./Age (years)	5.6	1	0.02 *
Raccoon dog	*Cryptosporidium* spp.	*G. duodenalis*	10.9	1	<0.001 ***
		Age (years)	0.1	1	0.7
		Forestry	0.8	2	0.7
	*G. duodenalis*	*Cryptosporidium* spp.	10.8	1	0.001 ***
		Age (years)	2.7	1	0.09

Abbreviations: LR Chisq—likelihood ratio chi-square test; Df—degrees of freedom; * *p* < 0.05; ** *p* < 0.01; *** *p* < 0.001.

## Data Availability

The original contributions presented in this study are included in the article/Supplementary Materials. Further inquiries can be directed to the corresponding author.

## Supplementary Materials

The following supporting information can be downloaded at: https://www.mdpi.com/article/10.3390/ani14233484/s1, File S1: Table with raw dataset.

## References

[1] Ryan U.M., Feng Y., Fayer R., Xiao L. (2021). Taxonomy and Molecular Epidemiology of *Cryptosporidium* and *Giardia*–A 50-Year Perspective (1971–2021). Int. J. Parasitol..

[2] Adam R.D. (2001). Biology of *Giardia lamblia*. Clin. Microbiol. Rev..

[3] Rosanowski S.M., Banica M., Ellis E., Farrow E., Harwood C., Jordan B., James C., McKenna D., Fox M., Blake D.P. (2018). The Molecular Characterisation of *Cryptosporidium* Species in Relinquished Dogs in Great Britain: A Novel Zoonotic Risk?. Parasitol. Res..

[4] Simonato G., Frangipane di Regalbono A., Cassini R., Traversa D., Tessarin C., Di Cesare A., Pietrobelli M. (2017). Molecular Detection of *Giardia duodenalis* and *Cryptosporidium* spp. in Canine Fecal Samples Contaminating Public Areas in Northern Italy. Parasitol. Res..

[5] Sotiriadou I., Pantchev N., Gassmann D., Karanis P. (2013). Molecular Identification of *Giardia* and *Cryptosporidium* from Dogs and Cats. Parasite.

[6] Cacciò S.M., Lalle M., Svärd S.G. (2018). Host Specificity in the *Giardia duodenalis* Species Complex. Infect. Genet. Evol..

[7] Thompson R.A., Olson M.E., Zhu G., Enomoto S., Abrahamsen M.S., Hijjawi N.S. (2005). *Cryptosporidium* and Cryptosporidiosis. Adv. Parasitol..

[8] Thompson R.A., Palmer C.S., O’Handley R. (2008). The Public Health and Clinical Significance of *Giardia* and *Cryptosporidium* in Domestic Animals. Vet. J..

[9] Hamnes I.S., Gjerde B.K., Robertson L.J. (2007). A Longitudinal Study on the Occurrence of *Cryptosporidium* and *Giardia* in Dogs During Their First Year of Life. Acta Vet. Scand..

[10] Taghipour A., Olfatifar M., Bahadory S., Godfrey S.S., Abdoli A., Khatami A., Javanmard E., Shahrivar F. (2020). The Global Prevalence of *Cryptosporidium* Infection in Dogs: A Systematic Review and Meta-Analysis. Vet. Parasitol..

[11] Murnik L.C., Daugschies A., Delling C. (2022). *Cryptosporidium* Infection in Young Dogs from Germany. Parasitol. Res..

[12] Szydłowicz M., Zajączkowska Ż., Lewicka A., Łukianowski B., Kamiński M., Holubová N., Sak B., Kváč M., Kicia M. (2024). Host-Specific *Cryptosporidium*, *Giardia*, and *Enterocytozoon bieneusi* in Shelter Dogs from Central Europe. Parasitology.

[13] Bouzid M., Halai K., Jeffreys D., Hunter P.R. (2015). The Prevalence of *Giardia* Infection in Dogs and Cats, a Systematic Review and Meta-Analysis of Prevalence Studies from Stool Samples. Vet. Parasitol..

[14] Claerebout E., Casaert S., Dalemans A.C., De Wilde N., Levecke B., Vercruysse J., Geurden T. (2009). *Giardia* and Other Intestinal Parasites in Different Dog Populations in Northern Belgium. Vet. Parasitol..

[15] Ferreira F.S., Pereira-Baltasar P., Parreira R., Padre L., Vilhena M., Tavira L.T., Atouguia J., Centeno-Lima S. (2011). Intestinal Parasites in Dogs and Cats from the District of Évora, Portugal. Vet. Parasitol..

[16] Drake J., Sweet S., Baxendale K., Hegarty E., Horr S., Friis H., Goddu T., Ryan W.G., von Samson-Himmelstjerna G. (2022). Detection of *Giardia* and Helminths in Western Europe at Local K9 (Canine) Sites (DOGWALKS Study). Parasites Vectors.

[17] Šmit I., Potočnjak D., Matijatko V., Torti M., Jović I., Grden D., Crnogaj M., Beck R. (2023). The Influence of *Giardia duodenalis* on the Occurrence of Clinical Signs in Dogs. Vet. Sci..

[18] Dubná S., Langrová I., Nápravník J., Jankovská I., Vadlejch J., Pekár S., Fechtner J. (2007). The Prevalence of Intestinal Parasites in Dogs from Prague, Rural Areas, and Shelters of the Czech Republic. Vet. Parasitol..

[19] Epe C., Rehkter G., Schnieder T., Lorentzen L., Kreienbrock L. (2010). *Giardia* in Symptomatic Dogs and Cats in Europe—Results of a European Study. Vet. Parasitol..

[20] Tysnes K.R., Skancke E., Robertson L.J. (2014). Subclinical *Giardia* in Dogs: A Veterinary Conundrum Relevant to Human Infection. Trends Parasitol..

[21] Debenham J.J., Landuyt H., Troell K., Tysnes K., Robertson L.J. (2017). Occurrence of *Giardia* in Swedish Red Foxes (*Vulpes vulpes*). J. Wildl. Dis..

[22] Hamnes I.S., Gjerde B.K., Forberg T., Robertson L.J. (2007). Occurrence of *Giardia* and *Cryptosporidium* in Norwegian Red Foxes (*Vulpes vulpes*). Vet. Parasitol..

[23] Hodžić A., Alić A., Omeragić J. (2014). Occurrence of *Cryptosporidium* spp. and *Giardia duodenalis* in Red Foxes (*Vulpes vulpes*) in Bosnia and Herzegovina. Maced. Vet. Rev..

[24] Osten-Sacken N., Słodkowicz-Kowalska A., Pacoń J., Skrzypczak Ł., Werner A. (2017). Intestinal and External Parasites of Raccoon Dogs (*Nyctereutes procyonoides*) in Western Poland. Ann. Parasitol..

[25] Mateo M., de Mingo M.H., de Lucio A., Morales L., Balseiro A., Espí A., Barral M., Barbero J.F.L., Habela M.Á., Fernández-García J.L. (2017). Occurrence and Molecular Genotyping of *Giardia duodenalis* and *Cryptosporidium* spp. in Wild Mesocarnivores in Spain. Vet. Parasitol..

[26] Onac D., Oltean M., Mircean V., Jarca A., Cozma V. (2015). Occurrence of *Giardia duodenalis* Zoonotic Assemblages in Red Foxes from Romania. Sci. Parasitol..

[27] Sullivan K.M., Dean A., Soe M.M. (2009). OpenEpi: A Web-Based Epidemiologic and Statistical Calculator for Public Health. Public Health Rep..

[28] Agricultural Data Centre Public Statistics of Pet Animals. https://registri.ldc.gov.lv/pub_istabas_dz/pub_istabas_dz.php.

[29] Harvey N.D. (2021). How Old Is My Dog? Identification of Rational Age Groupings in Pet Dogs Based Upon Normative Age-Linked Processes. Front. Vet. Sci..

[30] Ministry of Agriculture, Food and Veterinary Service (2021). Latvian State Program for the Control and Eradication of Rabies. https://www.pvd.gov.lv/lv/media/1109/download.

[31] Kuczynska E., Shelton D.R. (1999). Method for Detection and Enumeration of *Cryptosporidium parvum* Oocysts in Feces, Manures, and Soils. Appl. Environ. Microbiol..

[32] Maddox-Hyttel C., Langkjær R.B., Enemark H.L., Vigre H. (2006). *Cryptosporidium* and *Giardia* in Different Age Groups of Danish Cattle and Pigs—Occurrence and Management-Associated Risk Factors. Vet. Parasitol..

[33] QGIS.org. QGIS Geographic Information System; QGIS Association. http://www.qgis.org.

[34] R Core Team (2023). R: A Language and Environment for Statistical Computing.

[35] Sakamoto Y., Ishiguro M., Kitagawa G. (1986). Akaike Information Criterion Statistics; Mathematics and Its Applications.

[36] Fox J., Weisberg S. (2018). An R Companion to Applied Regression.

[37] Li J., Ryan U., Guo Y., Feng Y., Xiao L. (2021). Advances in Molecular Epidemiology of Cryptosporidiosis in Dogs and Cats. Int. J. Parasitol..

[38] Neves D., Lobo L., Simões P.B., Cardoso L. (2014). Frequency of Intestinal Parasites in Pet Dogs from an Urban Area (Greater Oporto, Northern Portugal). Vet. Parasitol..

[39] Chastant S., Mila H. (2019). Passive Immune Transfer in Puppies. Anim. Reprod. Sci..

[40] Day M.J. (2010). Ageing, Immunosenescence, and Inflammageing in the Dog and Cat. J. Comp. Pathol..

[41] Willems A., Paepe D., Marynissen S., Smets P., Van de Maele I., Picavet P., Duchateau L., Daminet S. (2017). Results of Screening of Apparently Healthy Senior and Geriatric Dogs. J. Vet. Intern. Med..

[42] Pipia A.P., Varcasia A., Tamponi C., Sanna G., Soda M., Paoletti B., Traversa D., Scala A. (2014). Canine Giardiosis in Sardinia Island, Italy: Prevalence, Molecular Characterization, and Risk Factors. J. Infect. Dev. Ctries..

[43] Remesar S., García-Dios D., Calabuig N., Prieto A., Díaz-Cao J.M., López-Lorenzo G., López C., Fernández G., Morrondo P., Panadero R. (2022). Cardiorespiratory Nematodes and Co-Infections with Gastrointestinal Parasites in New Arrivals at Dog and Cat Shelters in North-Western Spain. Transbound. Emerg. Dis..

[44] López-Arias Á., Villar D., López-Osorio S., Calle-Vélez D., Chaparro-Gutiérrez J.J. (2019). *Giardia* Is the Most Prevalent Parasitic Infection in Dogs and Cats with Diarrhea in the City of Medellín, Colombia. Vet. Parasitol. Reg. Stud. Rep..

[45] Adell-Aledón M., Köster P.C., de Lucio A., Puente P., Hernández-de-Mingo M., Sánchez-Thevenet P., Dea-Ayuela M.A., Carmena D. (2018). Occurrence and Molecular Epidemiology of *Giardia duodenalis* Infection in Dog Populations in Eastern Spain. BMC Vet. Res..

[46] Mateo M., Montoya A., Bailo B., Köster P.C., Dashti A., Hernández-Castro C., Saugar J.M., Matas P., Xiao L., Carmena D. (2023). Prevalence and Public Health Relevance of Enteric Parasites in Domestic Dogs and Cats in the Region of Madrid (Spain) with an Emphasis on *Giardia duodenalis* and *Cryptosporidium* sp.. Vet. Med. Sci..

[47] Barutzki D., Thompson R.C.A., Wielinga C., Parka U., Schaper R. (2007). Observations on *Giardia* Infection in Dogs from Veterinary Clinics in Germany. Parasitol. Res..

[48] Scorza A.V., Buch J., Franco P., McDonald C., Chandrashekar R., Lappin M.R. (2021). Evaluation for Associations Amongst *Giardia duodenalis* Assemblages and Diarrhea in Dogs. Vet. Parasitol..

[49] Day M.J., Horzinek M.C., Schultz R.D., Squires R.A. (2016). WSAVA Guidelines for the Vaccination of Dogs and Cats. J. Small Anim. Pract..

[50] Squires R.A., Crawford C., Marcondes M., Whitley N. (2024). 2024 Guidelines for the Vaccination of Dogs and Cats–Compiled by the Vaccination Guidelines Group (VGG) of the World Small Animal Veterinary Association (WSAVA). J. Small Anim. Pract..

[51] Barutzki D., Schaper R. (2013). Age-Dependent Prevalence of Endoparasites in Young Dogs and Cats up to One Year of Age. Parasitol. Res..

[52] Sommer M.F., Rupp P., Pietsch M., Kaspar A., Beelitz P. (2018). *Giardia* in a Selected Population of Dogs and Cats in Germany–Diagnostics, Coinfections and Assemblages. Vet. Parasitol..

[53] Leoni F., Amar C., Nichols G., Pedraza-Diaz S., McLauchlin J. (2006). Genetic Analysis of *Cryptosporidium* from 2414 Humans with Diarrhoea in England Between 1985 and 2000. J. Med. Microbiol..

[54] Lucio-Forster A., Griffiths J.K., Cama V.A., Xiao L., Bowman D.D. (2010). Minimal Zoonotic Risk of Cryptosporidiosis from Pet Dogs and Cats. Trends Parasitol..

[55] Matos M., Alho A.M., Owen S.P., Nunes T., Madeira de Carvalho L. (2015). Parasite Control Practices and Public Perception of Parasitic Diseases: A Survey of Dog and Cat Owners. Prev. Vet. Med..

[56] Overgaauw P.A., Van Zutphen L., Hoek D., Yaya F.O., Roelfsema J., Pinelli E., Van Knapen F., Kortbeek L.M. (2009). Zoonotic Parasites in Fecal Samples and Fur from Dogs and Cats in The Netherlands. Vet. Parasitol..

[57] Bajer A., Bednarska M., Rodo A. (2011). Risk Factors and Control of Intestinal Parasite Infections in Sled Dogs in Poland. Vet. Parasitol..

[58] Mircean V., Györke A., Cozma V. (2012). Prevalence and Risk Factors of *Giardia duodenalis* in Dogs from Romania. Vet. Parasitol..

[59] Hubbard K., Skelly B.J., McKelvie J., Wood J.L.N. (2007). Risk of Vomiting and Diarrhoea in Dogs. Vet. Rec..

[60] Stavisky J., Radford A.D., Gaskell R., Dawson S., German A., Parsons B., Clegg S., Newman J., Pinchbeck G. (2011). A Case–Control Study of Pathogen and Lifestyle Risk Factors for Diarrhoea in Dogs. Prev. Vet. Med..

[61] Deksne G., Mateusa M., Cvetkova S., Derbakova A., Keidāne D., Troell K., Schares G. (2022). Prevalence, Risk Factor and Diversity of *Cryptosporidium* in Cattle in Latvia. Vet. Parasitol. Reg. Stud. Rep..

[62] Cardoso A.S., Costa I.M.H., Figueiredo C., Castro A., Conceição M.A.P. (2014). The Occurrence of Zoonotic Parasites in Rural Dog Populations from Northern Portugal. J. Helminthol..

[63] Beck R., Sprong H., Lucinger S., Pozio E., Caccio S.M. (2011). A Large Survey of Croatian Wild Mammals for *Giardia duodenalis* Reveals a Low Prevalence and Limited Zoonotic Potential. Vector-Borne Zoonotic Dis..

[64] Barrera J.P., Carmena D., Rodríguez E., Checa R., López A.M., Fidalgo L.E., Gálvez R., Marino V., Fuentes I., Miró G. (2020). The Red Fox (*Vulpes vulpes*) as a Potential Natural Reservoir of Human Cryptosporidiosis by *Cryptosporidium hominis* in Northwest Spain. Transbound. Emerg. Dis..

[65] Adriana G., Zsuzsa K., Oana D.M., Mircea G.C., Viorica M. (2016). *Giardia duodenalis* Genotypes in Domestic and Wild Animals from Romania Identified by PCR-RFLP Targeting the gdh Gene. Vet. Parasitol..

[66] Perec-Matysiak A., Hildebrand J., Popiołek M., Buńkowska-Gawlik K. (2023). The Occurrence of *Cryptosporidium* spp. in Wild-Living Carnivores in Poland—A Question Concerning Its Host Specificity. Pathogens.

[67] Solarczyk P., Majewska A.C., Jędrzejewski S., Górecki M.T., Nowicki S., Przysiecki P. (2016). First Record of *Giardia* Assemblage D Infection in Farmed Raccoon Dogs (*Nyctereutes procyonoides*). Ann. Agric. Environ. Med..

[68] Soulsbury C.D., Iossa G., Baker P.J., Cole N.C., Funk S.M., Harris S. (2007). The Impact of Sarcoptic Mange *Sarcoptes scabiei* on the British Fox *Vulpes vulpes* Population. Mammal Rev..

